# Cardiopulmonary Exercise Test in Heart Failure and the Benefits of Exercise and Rehabilitation—An Updated Review

**DOI:** 10.31083/RCM44598

**Published:** 2025-09-26

**Authors:** Stefanos G. Sakellaropoulos, Andreas Mitsis

**Affiliations:** ^1^Department of Cardiology, University Hospital and University of Basel, 4001 Basel, Switzerland; ^2^Department of Cardiology, Nicosia General Hospital, 1010 Nicosia, Cyprus

## 1. Heart Failure Prognosis 

Several studies confirm the power of peak Oxygen Consumption VO_2_ in terms of Prognosis [1, 2, 3]. Weber and Janicki [4] introduced the poor correlation between left ventricular ejection fraction (LVEF), peak VO_2_, wedge pressure and cardiac index [5].

Another study from Matsumura *et al*. [6] showed that the New York Heart Association (NYHA) classification can be correlated with the anaerobic threshold (AT) and peak VO_2_. In addition, studies from Weber and Janicki demonstrate a more objective correlation between symptoms and peak VO_2_ and AT.

In the association between VO_2_/kg, the A-E Classification appears to be superior to the NYHA Classification [4]. A consensus document agreed with this assessment [7].

## 2. Exercise Oscillatory Ventilation

Approximately 30 years ago, Guazzi *et al*. [8] showed that exercise oscillatory ventilation (EOV) is considered as a risk factor for heart failure patients with reduced ejection fraction in terms of morbidity and mortality, with a prevalence of 30%. The same group demonstrated the same prevalence in patients with preserved ejection fraction. Using cardiopulmonary exercise testing, Sakellaropoulos *et al*. [9] demonstrated that there is a prevalence of 5% in patients with hypertrophic cardiomyopathy [10].

Corrà *et al*. [11] found that EOV is not present in healthy individuals, but was observed only in cardiovascular disease (CVD) patients and in those with depressed LVEF, with a prevalence of 1.9% with a LVEF of 41–49%, a prevalence of 3.4% with LVEF ≤40% and 7.3% in severe heart failure. A subanalysis of the EURO-EX trial [8] demonstrated that the EOV for females and diabetes was 3.71, reflecting an imminent cardiovascular event.

## 3. Left Ventricular Assist Device (LVAD)—Implantation and Explantation

The criteria for Implantation are defined from the REMATCH and Heart mate II studies. These include patients who are not candidates for heart transplantation, significant functional limitations with chronic NYHA IV symptoms for 45 of 60 days despite use of optimal medical therapy, LVEF less than 25%, and peak VO_2_ of 14 mL/kg/min or less [12].

Criteria for LVAD explantation include peak VO_2_, as well as filling pressures and cardiac output. These parameters include an ejection fraction of less than 45%, left ventricular end diastolic diameter <60 mm, cardiac index >2.8 L/min/m^2^, PAWP <12 mmHg, a ventilation (VE)/VCO_2_ of <34 during low LVAD speed testing or a peak VO_2_ less more than 16 mL/kg/min [13] (Table 1).

**Table 1.** **Parameters of CPET**.

**Panels Information**
Panel 1 VE and load against time
Panel 2 HR and O_2_-pulse against time
Panel 3 VO_2_, VCO_2_, and load against time
Panel 4 VE against VCO_2_
Panel 5 HR and VCO_2_ against VO_2_
Panel 6 EqO_2_ and EqCO_2_ against time
Panel 7 VTex against VE
Panel 8 RER and BR FEV% against tune
Panel 9 PETO_2_ and PETCO_2_ as well as PaO_2_ and PaCO_2_ against time
**CPET Parameters for heart failure**
Peak VO_2_ (mL/kg/min)
Maximal Oxygen Consumption
VE/VCO_2_ Slope
Ventilatory efficiency, normal <30
O_2_ Puls (mL/Heartbeat)
PetCO_2_ (mmHg)
End-tidal CO_2_, normal >30
Peak RER

VE, Ventilation; HR, Heart Rate; RER, Respiratory exchange Ratio; BR, Breath Rate; CPET, Cardiopulmonary Exercise Test; FEV, Forced expiratory Volume.

Imamura *et al*. [14] demonstrated in a Cox regression analysis that after implantation of an LVAD, a Cox E1 (maximum load 51W), E2 (minute ventilation/carbon dioxide output [V̇ E/V̇ CO_2_] slope and E3 (peak oxygen consumption [PV̇ O_2_] 12.8 mL/kg/min could predict explantation in the course 2 years (*p* < 0.05 for all). The sum of positive E1-3 significantly stratified the 2-year cumulative explantation rate into low (0 points), intermediate (1–2 points), and high (3 points) expectancy groups (0%, 29%, and 86%, respectively, *p* < 0.001).

## 4. Assessment of Effects of Exercise Training and Rehabilitation in LVAD Patients Based on Peak VO_2_

Grosman-Rimon *et al*. [15] showed that rehabilitation led to increased peak VO_2_ values, and improvement in the 6-minute walk test. Furthermore, no significant differences in VE/VCO_2_ or AT have been demonstrated. Therefore, Exercise Rehabilitation is highly recommended in LVAD patients [15].

## 5. Exercise Effects and Evaluation of Patients for Heart Transplantation

The landmark study of Stevenson [16] reviewed data on 68 heart failure patients, listed for transplantation. All patients repeated exercise tests at a mean 6 +/– months. All 68 patients were treated with the maximal tolerated heart failure treatment and reduction of cardiac afterload, as well as defined and personalized exercise rehabilitation training.

38 patients showed an improved peak VO_2_ >2 mL/kg/min, with a total value of more than 12 mL/kg/min. 31 patients experienced a significant improvement in their clinical condition, characterized by a significant improvement of peak O_2_-Pulse, AT, and heart rate reserve. The was also a reduction in resting heart rate. From the initial 68 patients, 45% have been removed from the transplantion list. The survival rate was 100% [16].

## 6. Contraindications of CPET and Clinical Communication Points

There are specific contraindications of cardiopulmonary exercise testing (CPET). An acute myocardial infarction within 2 to 3 days, active myo- or endocarditis and uncontrolled symptomatic, and decompensated heart failure are considered absolute contraindications. Furthermore, in terms of acute coronary syndromes, unstable angina not previously stabilized by medical therapy is also a contraindication. Moreover, patients with uncontrolled, hemodynamic relevant cardiac arrhythmias should never undergone CPET. Finally, in terms of valvulopathy, severe aortic stenosis is considered an absolute contraindication. Non-cardiac contraindications include acute pulmonary edema, acute respiratory failure, advanced complicated pregnancy, and severe uncorrected electrolyte abnormalities [17].

Cardiologists, Pulmonologists or expert physicians should discuss these results in patients and family physicians. In patients with heart failure, CPET provides information about the cardiac, pulmonary and musculoskeletal performance, in combination with laboratory results, to exclude conditions such as anemia or hyperthyroidism, that alter the hemodynamic status of patients [18].

CPET is an excellent tool for therapy decision making and follow-up due to its synergistic prognostic and predictive power. Under these terms, all of the cardiovascular medical therapies can be monitored, and individually adjusted, to be used for their efficacy and clinical outcomes [18]. These exercise protocols can be individualized for each patient, in combination with vital parameters, to improve patient outcomes. For example, aerobic, adjusted exercise can influence and improve VO_2_ in patients with terminal heart failure, an improvement that can lead to improved prognosis and ultimately avoiding heart transplantation as a destination therapy [18].

## 7. Conclusion and Future Aspects

Cardiopulmonary exercise testing in combination with circulating metabolites and mRNAs can contribute to the diagnosis of the initial stages of heart failure. CPET can help to determine the diagnosis of many cardiac diseases and can assist in refining the severity of heart failure, assessment of risk stratification, and ultimately can be used for evaluation of heart transplantation, and implantation as well as explantation of left ventricular assist devices.

## Abbreviations

LVAD, Left ventricular assist device; LVEF, Left ventricular ejection fraction; EOV, Exercise oscillatory ventilation; CPET, Cardiopulmonary exercise testing.
